# Molecular evolution of juvenile hormone esterase-like proteins in a socially exchanged fluid

**DOI:** 10.1038/s41598-018-36048-1

**Published:** 2018-12-13

**Authors:** Adria C. LeBoeuf, Amir B. Cohanim, Céline Stoffel, Colin S. Brent, Patrice Waridel, Eyal Privman, Laurent Keller, Richard Benton

**Affiliations:** 1 Department of Physics of Complex Systems, Weizmann Institute of Science, Rehovot, Israel; 2 Center for Integrative Genomics, University of Lausanne, Lausanne, Switzerland; 3 Department of Ecology and Evolution, University of Lausanne, Lausanne, Switzerland; 4 Department of Evolutionary and Environmental Biology, Institute of Evolution, University of Haifa, Haifa, Israel; 5 Arid Land Agricultural Research Center, USDA-ARS, Maricopa, United States; 6 Protein Analysis Facility, University of Lausanne, Lausanne, Switzerland

## Abstract

Socially exchanged fluids are a direct means by which an organism can influence conspecifics. It was recently shown that when workers of the carpenter ant *Camponotus floridanus* feed larval offspring via trophallaxis, they transfer Juvenile Hormone III (JH), a key developmental regulator, as well as paralogs of JH esterase (JHE), an enzyme that catalyzes the hydrolysis of JH. Here we combine proteomic, phylogenetic and selection analyses to investigate the evolution of this esterase subfamily. We show that *Camponotus* JHE-like proteins have undergone multiple duplications, experienced positive selection, and changed tissue localization to become abundantly and selectively present in trophallactic fluid. The *Camponotus* trophallactic esterases have maintained their catalytic triads and contain a number of positively-selected amino acid changes distributed throughout the protein, which possibly reflect an adaptation to the highly acidic trophallactic fluid of formicine ants. To determine whether these esterases might regulate larval development, we fed workers with a JHE-specific pharmacological inhibitor to introduce it into the trophallactic network. This inhibitor increased the likelihood of pupation of the larvae reared by these workers, similar to the influence of food supplementation with JH. Together, these findings suggest that JHE-like proteins have evolved a new role in the inter-individual regulation of larval development in the *Camponotus* genus.

## Introduction

Coordination between cells in a multicellular organism often occurs through hormones, which bind to receptors on or in different cell types throughout the body. Analogously, coordination between individuals in insect colonies is frequently mediated by chemical communication (i.e., pheromones). Many social insects also engage in oral trophallaxis, a mouth-to-mouth fluid transfer that connects every member of the colony, including larvae. Trophallaxis was previously assumed to be mainly a food-sharing mechanism^1–3^. However, we recently showed that trophallactic fluid in *Camponotus floridanus* carpenter ants contains hormones, nestmate recognition cues, small RNAs, and a variety of proteins, many of which have been associated with growth and development^4^, suggesting broader functions for this fluid in inter-individual communication. Amongst these molecules, the presence of Juvenile Hormone III (JH) was of particular interest^4^ because this hormone is a key regulator of insect development^5,6^ and reproduction^7–9^ across insects, and of caste determination^5–7,10–13^ and division of labor^14–18^ in social insects. During larval development, JH works in conjunction with another group of hormones, the ecdysteroids, to induce successive molts and pupation and determine developmental trajectory^10,11,19–21^.

JH levels are modulated through varied rates of biosynthesis and degradation. In canonical insect physiology, JH is synthesized by paired corpora allata in both larval and adult stages^6^. After being released into the hemolymph, JH is primarily degraded by two enzymes that peak in late-larval development, JH esterase (JHE), which is produced in the fat body, and the more broadly-expressed, membrane-bound JH epoxide hydrolase^22,23^. Intriguingly, in addition to JH, *C*. *floridanus* trophallactic fluid contains abundant JHE-like proteins^4^, raising questions about the role of these enzymes in this fluid.

## Results

### Variable presence of JH and JHE-like proteins in social insect trophallactic fluid

We first assessed whether JH and JHEs are present in the trophallactic fluid of three additional species (*Camponotus fellah*, *Solenopsis invicta* (red imported fire ant) and *Apis mellifera* (European honey bee)), by reanalyzing previous data^4^ and combining this with additional small-molecule and proteomic analyses (Materials and Methods). The trophallactic fluids of both *Camponotus* species contain JH (Supplementary Table S1) and abundant peptides mapping to multiple JHE-like proteins (approximately 18% of total trophallactic fluid protein in both species, Supplementary Table S2). By contrast, the trophallactic fluids of *S*. *invicta* and *A*. *mellifera* contain neither the hormone nor JHE orthologs; the esterases we do detect are very distant in sequence to JHEs (Supplementary Tables S1 and S2). These results indicate that the presence of JH and JHEs in this social fluid is variable across social insects, implying that it is unlikely to simply result from a passive process such as diffusion from hemolymph.

### The esterase repertoire of *C. floridanus*

**Figure 1:**
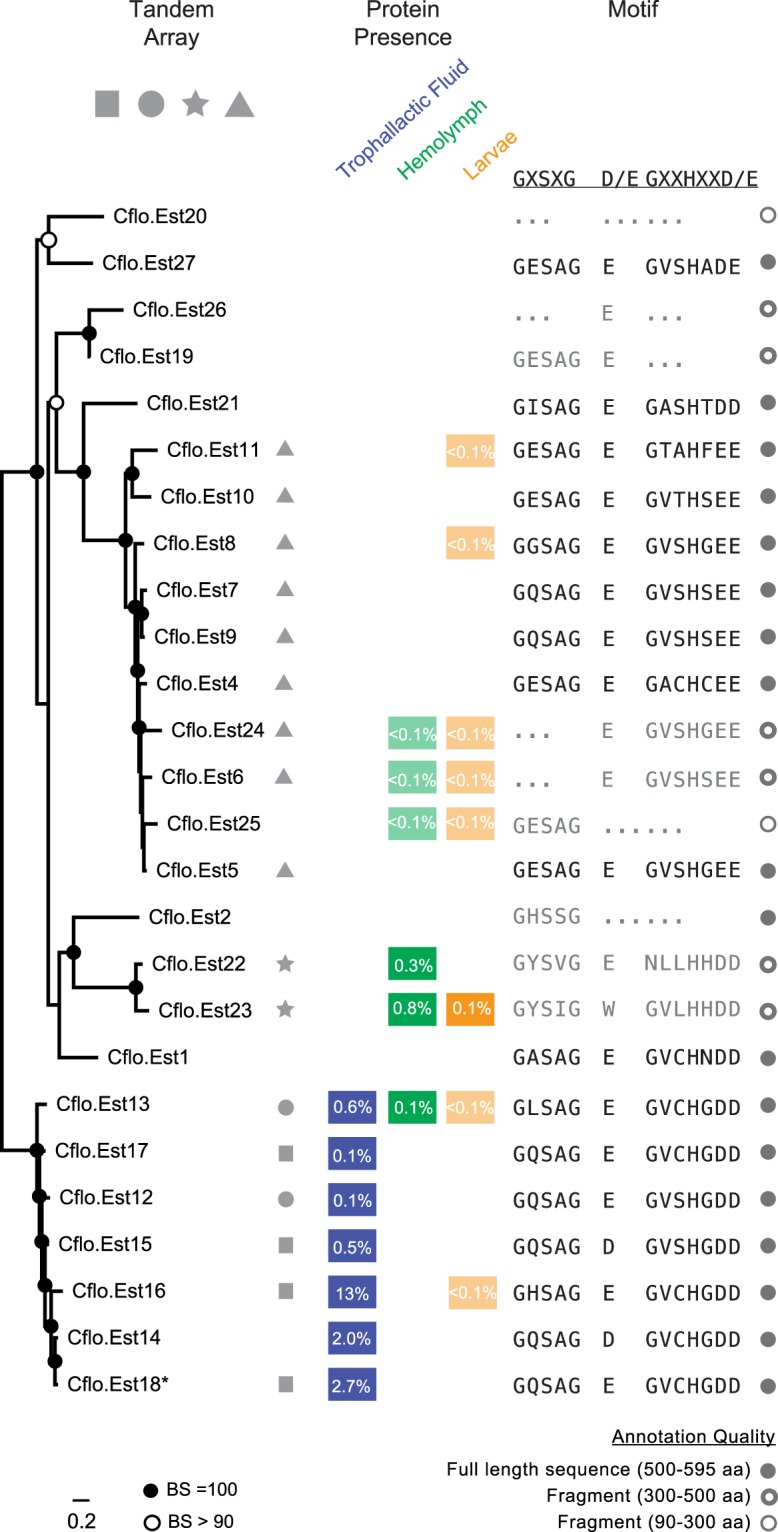
The esterase repertoire in *C*. *floridanus*. Maximum likelihood tree of re-annotated *C*. *floridanus* carboxylesterases. Bootstrap (BS) values greater than 90 and equal to 100 are indicated by empty circle nodes and black nodes, respectively. The four tandem arrays of esterases observed in the genome are represented by symbols. Cflo.Est14 is on a single-gene scaffold; circle and square arrays are found on the edge of contigs, indicating that these seven esterases may be a continuous DNA segment. Protein abundance is also shown in Supplementary Table S2 (percent of total NSAF). Catalytic triad sequence motifs in each carboxylesterase gene are shown in black if all motifs are present, and are shown in bold if the specific amino acids are consistent with known JHEs. Sequences are shown in grey if one or more residues of the catalytic triad are missing; ellipses indicate missing sequence regions. Sequence length of each esterase is shown in grey circles, where filled circles indicate a complete or nearly complete sequence.

Insect genomes encode many esterases with diverse substrates, yet any given species has one active JHE, defined by its ability to efficiently catalyze the hydrolysis of JH to JH acid either *in vitro* or *in vivo*, and by its expression in late larval development and in the adult fat body^22–30^. Our proteomic survey of *C*. *floridanus*^4^ revealed that hemolymph and trophallactic fluid contain different JHE-like proteins. To explore the evolutionary relationships of these enzymes, we first re-annotated all carboxylesterases within the *C*. *floridanus* genome, identifying 26 genes and gene fragments (Fig. 1, Supplementary File 1). Although the *C*. *floridanus* genome is incompletely assembled (v3.3; 10% of base pairs are found in scaffolds under 4 kbp), we noted that 17 of 26 esterase genes localize to four tandem arrays (Fig. 1), indicative of recent tandem duplications. Two of these arrays and one single-gene scaffold encode the seven JHE-like proteins detected in trophallactic fluid.

To assess these esterases’ potential for JHE enzymatic function, we checked for the presence of the sequence motifs of the catalytic triad (GxSxG, E/D, GxxHxxD/E)^26,31^. The serine of the catalytic triad for functionally characterized JHEs lies within a GQSAG motif, while other non-JH-specific esterases have different motifs (e.g., GESAG in *D*. *melanogaster*’s α-esterases). In *C*. *floridanus*, all of the esterases present in trophallactic fluid have the amino acid motifs necessary to catalyze the hydrolysis of JH, while many esterases present in hemolymph and in larvae do not (Fig. 1).

### *Camponotus* trophallactic esterases have duplicated, experienced positive selection and become abundant in trophallactic fluid

To gain insight into the evolution of the *C*. *floridanus* trophallactic esterases, we re-annotated carboxylesterase genes of 33 ant species whose genomes or transcriptomes were available (i.e., the 19 available *Formicinae*, including six *Camponotus* species and eight species of the comparably species-rich formicine genus *Formica*, 11 *Myrmicinae*, and three more distantly related species, Supplementary Files 1 and 2). We similarly re-annotated carboxylesterase genes in four outgroups (*A*. *mellifera*, *Nasonia vitripennis* (parasitoid jewel wasp), *Culex quinquefasciatus* (mosquito), *Manduca sexta* (hawkmoth)) and constructed an initial protein tree of all the esterases (Supplementary Fig. S3) that allowed us to define membership in the clade containing the trophallactic esterases. To place the *Camponotus* trophallactic esterases in the broader context of previously characterized JHEs and other esterases within the carboxylesterases gene family, we built a protein tree including the re-annotated esterase sequences longer than 400 amino acids from six ant genomes, five outgroups and five JHEs from other insect orders (Supplementary Fig. S4). Consistent with previous work^30,32^, JHEs assorted phylogenetically into four clusters. Notably, the *C*. *floridanus* trophallactic esterases are found within a clade stemming from the only known *A*. *mellifera* JHE (Amel.Est1), indicating that in spite of their different localization (trophallactic fluid vs. hemolymph), these esterases may perform similar JH-regulatory functions.

**Figure 2:**
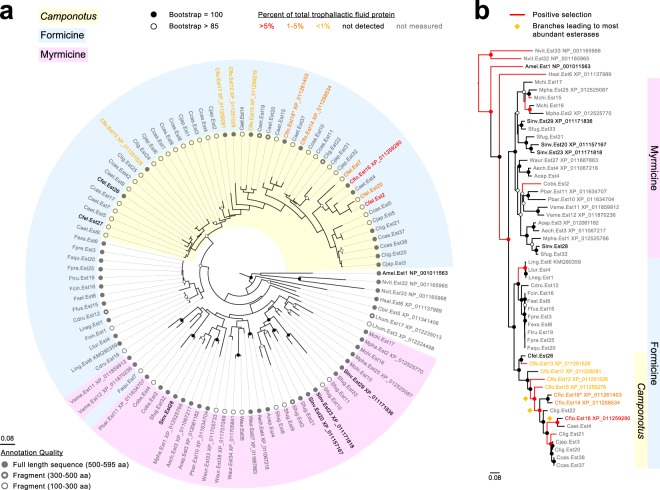
*Camponotus* trophallactic esterases have duplicated, experienced positive selection and increased in trophallactic fluid abundance. Protein trees of esterases in the clade containing the *Camponotus* trophallactic esterases (corresponding to the highlighted subtree in Supplementary Fig. S3), either for sequences longer than 100 amino acids (**a**) or 400 amino acids (**b**). For (**b**), red branches indicate positive selection (FDR < 0.1). Branch numbers and significance values can be found in Supplementary Fig. S6 and Table S5. Nodes with bootstrap values greater than 85 and equal to 100 are shown in empty circles and filled circles, respectively. The portion of the tree corresponding to formicine ants is indicated in light blue, and the sequences from the genus *Camponotus* in yellow. For the four species where trophallactic fluid protein abundance has been measured, sequence names are in bold and color-coded by protein abundance (specific percentages can be found in Supplementary Table S2). Branch length is based on amino acid changes. (**a**) Sequence length of each of the 101 esterase sequences from 31 species of ants, *A*. *mellifera* and *N*. *vitripennis* is shown in grey circles, where filled circles indicate a complete or nearly complete sequence. (**b**) Gold diamonds mark the three positively selected branches within which specific positively-selected sites are highlighted in Fig. 3.

To analyze duplication and positive selection of the *C*. *floridanus* trophallactic esterases, we performed a phylogenetic analysis of the subset of esterase sequences that fall within the clade for which the *A*. *mellifera* JHE is most basal (Fig. 2a). Multiple gene duplication events led to an expansion in the *Camponotus* clade, whereas no such repeated duplications were apparent in the sister clade of formicine esterases from *Formica*, *Lasius* and *Cataglyphis*. Each *Camponotus* species had more esterases in this clade than each species of *Formica* (5–12 vs 1–2; t-test, p < 10^−5^). To infer positive selection on the protein coding sequences, we applied the branch-site test for positive selection^33,34^, where each branch is tested for codon sites with a dN/dS ratio > 1. Because many of the re-annotated sequences were fragments due to relatively low genome and transcriptome quality in some species, we performed the positive selection analyses using only sequences longer than 400 amino acids (i.e., 54 out of the 101 sequences shown in Fig. 2a). We observed significant positive selection in 20 branches out of 109 (at FDR < 0.1, Fig. 2b, Supplementary Table S5, Fig. S6). Comparing *Camponotus* subtree with the subtree of other Formicine sequences, we observe positive selection in 30% vs 4% of the branches (χ^2^ = 5.38, p < 0.02). Thus, positive selection and associated duplications are apparent in this protein family in the *Camponotus* lineage.

Combining proteomic localization information with our phylogenetic analysis revealed that branch length (amino acid change) was positively correlated with protein presence in trophallactic fluid (Fig. 2a, Supplementary Fig. S7, Spearman’s rank correlation p < 0.0005, ρ = 0.85). The most basal *C*. *floridanus* esterase in this clade, Cflo.Est13, exhibits a low rate of evolution and a localization pattern (adult worker hemolymph, larvae, and trophallactic fluid) that is more typical of a JHE, suggesting conservation of an ancestral function.

### Positively selected amino acid changes may alter the function of abundant trophallactic esterases

**Figure 3:**
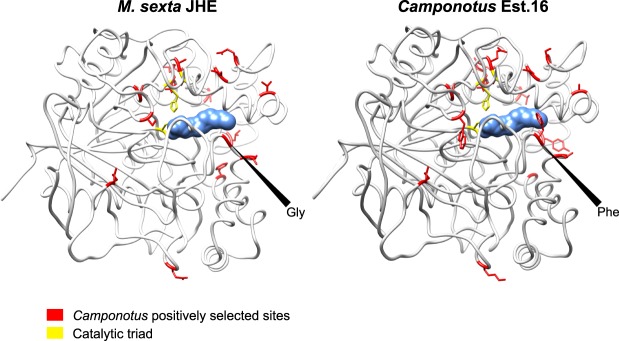
Positively selected amino acid changes in the abundant trophallactic esterases. The protein structure on the left is the lepidopteran *M*. *sexta* JHE (2JF0) crystallized with the JHE inhibitor OTFP (blue) covalently bound in the binding pocket. The *Camponotus* protein, on the right, is the same structure with the amino acids replaced at sites under positive selection (posterior probability >0.95, shown in red) in the three significant branches that differentiate the derived *Camponotus* sequences from the more ancestral sequences in *Camponotus* and *Formica*. At the mouth of the binding pocket, *M*. *sexta* has glycine at 2FJ0 position 312, *Formica* and Cflo.Est13 have proline, and Cflo.Est16 has phenylalanine (see Supplementary File 3). The catalytic triad is shown in yellow, with the exception of the D/E (*M*. *sexta* numbering: amino acid 357) shown with backbone in yellow and sidechain in red to indicate positive selection.

To better understand the link between positive selection and function, we analyzed the positively selected sites identified by the branch-site test (on sequences longer than 400 amino acids, posterior probability >0.95, Supplementary File 3) that were found on the branches with significant signatures of positive selection. In the three positively selected branches leading to the most abundant *Camponotus* esterases (marked with gold diamonds in Fig. 2b) we found 16 significantly positively-selected sites, and mapped these amino acids onto the crystal structure of the *M*. *sexta* JHE (2FJ0, Fig. 3)^22,26^. Most (14/16) of the positively-selected sites are located around the ligand binding region, including one member of the catalytic triad (the D/E at *M*. *sexta* JHE amino acid 357) and between the catalytic triad motifs and the exterior of the protein (Fig. 3). In only the most abundant trophallactic esterases, the outer lobe of the binding pocket is impinged upon by a phenylalanine (mutated from the more ancestral glycine in *M*. *sexta* or proline in *Formica* or Cflo.Est13; Fig. 3, Supplementary File 3). This region of the binding pocket corresponds to where the epoxide moiety of JH would lie^22,26^.

### JHE-inhibitor influences development *in vivo*

We next focused on the potential role of the trophallactic esterases in larval development. Many ant species exhibit temporally irregular development, where the time from egg-hatching to pupation varies widely, presumably because of differential feeding, even in the same batch of eggs^35–39^. We previously showed that additional JH in the trophallactic fluid of rearing *C*. *floridanus* workers increased the proportion of larvae successfully completing metamorphosis. The trophallactic JHE-like proteins present a possible means for rearing workers to regulate the presence of JH in trophallactic fluid and thus influence larval development.

**Figure 4:**
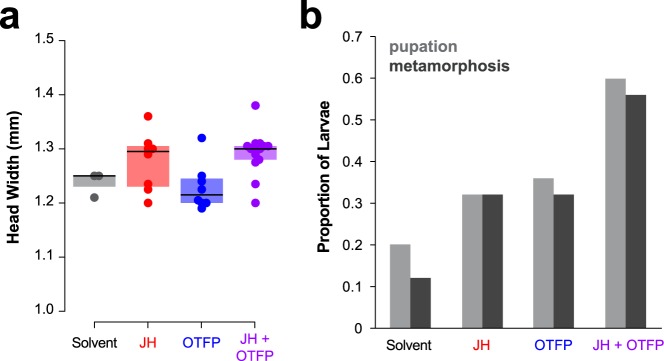
The trophallactic esterases influence development *in vivo*. (**a**) Head width of pupae raised by workers who were fed food supplemented with JH, OTFP, both JH and OTFP, or solvent alone. Median values and interquartile ranges are shown. GLMM testing for the effect of the two treatments on head width with colony as a random factor, JH, p < 0.001; OTFP, not significant. (**b**) Proportion of total larvae that pupated and the proportion of total larvae that metamorphosed when reared by workers that were fed food supplemented with JH, OTFP, both, or solvent only. Binomial GLMM testing effect of JH and OTFP on survival past metamorphosis with colony as a random factor, p < 0.018 for each of JH and OTFP. Two-way ANOVA yielded p < 0.017 for each treatment and there was no interaction between treatments. Source data available in Supplementary File 4. (**a**,**b**) contain information from the same experiment which contained five replicates per treatment where a replicate is 20–30 workers rearing five larvae over seven weeks.

Because all of the trophallactic esterases have an intact catalytic triad, their activity is likely to be blocked by the JHE inhibitor and transition-state-analog of JH, 3-octylthio-1,1,1-trifluoropropan-2-one (OTFP)^26,27,40^. We fed JH, OTFP, JH and OTFP together, or solvent alone to groups of worker ants rearing larvae. For each replicate, we measured head width of metamorphosed larvae as a proxy for body size, the number of larvae that formed a pre-pupa, and of these, the number that underwent metamorphosis. As observed previously, JH supplementation significantly increased head width (GLMM, JH and OTFP as fixed effects, and colony as random factor, p < 0.001, Fig. 4a) and increased the number of larvae that survived past metamorphosis (GLMM binomial, JH and OTFP as fixed effects, and colony as random factor p < 0.018, Fig. 4b). Treatment with OTFP resulted in no significant change in head width but increased the number of larvae that survived past metamorphosis (GLMM binomial p < 0.018). The two treatments independently influenced the proportion of larvae surviving metamorphosis (two-way ANOVA, p < 0.017 each for JH and OTFP; interaction not significant).

## Discussion

Our results reveal the evolution of a subfamily of JHE-like proteins in carpenter ants whose members have sustained many duplications, significant positive selection and shifted their location from hemolymph to the trophallactic fluid. These esterases have all maintained the catalytic triad characteristic of JHEs, and we speculate that some of the positively-selected sequence changes observed in these proteins reflect adaptation during their shift from hemolymph (pH 8) to the extreme acidity of Formicine trophallactic fluid (pH 2–4)^41,42^. Addition of a JHE inhibitor to the trophallactic fluid of rearing workers increased the proportion of larvae brought to pupation, similar to supplementation with JH. These observations – together with the conspicuous absence of such JHE-like proteins and JH in the trophallactic fluid of two other insect species – suggest that this enzyme subfamily has evolved to regulate JH levels in this socially-transmitted fluid.

The identification of seven distinct JHE-like proteins in *C*. *floridanus* trophallactic fluid indicates that this subfamily has multiple roles. For example, some may directly degrade JH, some might have JH-independent functions (e.g., in digestion of food components or in detoxification) and others could be pseudo-enzymes that protect – rather than degrade – JH as it is passed to larvae through the harsh trophallactic fluid environment. The selective presence of these JHE-like esterases in trophallactic fluid may provide a means to regulate the amount of JH transmitted over the social network independently of the levels circulating in an individual’s hemolymph. This would be a necessary transition for an individual-level developmental regulator to become a colony-level regulator.

## Materials and Methods

### Insect source and rearing

*C*. *floridanus* workers came from four colonies established in the laboratory from approximately 1-year-old founding queens and associated workers collected from the Florida Keys (Fiesta, Duck and Sugarloaf Keys) in 2006 and 2011. Ants were provisioned once a week with fresh sugar water, an artificial diet of honey or maple syrup, eggs, agar, canned tuna and a few *Drosophila melanogaster*. Maple syrup was substituted for honey and no *Drosophila* were provided in development and proteomic experiments to avoid contamination with other insect proteins. Colonies were maintained at 25 °C with 60% relative humidity and a 12 hr light:12 hr dark cycle. *C*. *fellah* workers came from three colonies established from queens collected after a mating flight in March 2007 in Tel Aviv, Israel (Colonies #5, 28, 33). The ant colonies were maintained at 32 °C with 60% relative humidity and a 12 hr light:12 hr dark cycle. Fire ant workers (*Solenopsis invicta*) were collected from three different colonies, two polygyne, one monogyne, maintained at 32 °C with 60% relative humidity and a 12 hr light:12 hr dark cycle. Honeybee workers (*A*. *mellifera*, Carnica and Buckfast) were collected from three different hives maintained with standard beekeeping practices.

### Sample collection

As described previously^4^, ants were anesthetized by CO_2_ (on a CO_2_ pad; Flypad, FlyStuff) and placed ventral-side up. The abdomen of each ant was lightly squeezed with soft forceps to prompt the regurgitation of fluids from the crop. Ants that underwent anesthesia and light squeezing recovered in approximately 5 min. Trophallactic fluid was collected with graduated borosilicate glass pipettes, and transferred immediately to pure EtOH for GC-MS measurement. Trophallactic fluid was pooled from groups of individuals from different colonies of *C*. *floridanus* (12–19 individuals, 1–12 µl per pooled group), *C*. *fellah* (16–46 individuals, 10–12 µl), *S*. *invicta* (39–137 individuals, 1–9 µl) and *A*. *mellifera* (5–20 individuals, 11–35 µl). Trophallactic fluid was collected from *S*. *invicta* in the same manner as from *C*. *floridanus* and *C*. *fellah*; for *S*. *invicta* more ants were used due to their smaller body size and crop volume. *A*. *mellifera* trophallactic fluid was collected from workers that were first cold-anesthetized and then transferred to a CO_2_ pad to ensure continual anesthesia during collection as described above. In each case, trophallactic fluids were pooled only from individuals of the same colony. Larvae were collected for proteomic measurement (three second-instar larvae for one sample and one fourth-instar larva for the other), sliced into 2–5 pieces with iris scissors, placed in an Eppendorf tube in buffer (100 mM NaH_2_PO_4_, 1 mM EDTA, 1 mM DTT, pH 7.4) and ground with an Eppendorf pestle. Samples were centrifuged for one minute to remove solids.

### JH quantification by GC-MS

As described previously^4^, for each sample, a known quantity of trophallactic fluid was collected into a graduated glass capillary tube and blown into an individual glass vial containing 5 µl of 100% ethanol. This biological sample was added to a 1:1 mixture of isooctane and methanol, processed and stored at –80 °C until analysis. Before analysis, 50% acetonitrile (HPLC grade) was added. Prior to purification, farnesol (Sigma-Aldrich, St Louis, MO) was added to each sample to serve as an internal standard. Briefly, samples were derivatized in a solution of methyl-d alcohol (Sigma-Aldrich, St Louis, MO) and trifluoroacetic acid (Sigma-Aldrich) then analyzed using an HP 7890 A Series GC (Agilent Technologies, Santa Clara, CA) coupled to an HP 5975 C inert mass selective detector monitoring at m/z 76 and 225 to ensure specificity for the d3-methoxyhydrin derivative of JH III. Total abundance was quantified against a standard curve of derivatized JH III, and adjusted for the starting volume of trophallactic fluid. The detection limit of the assay is approximately one pg.

### Proteomic analyses

Proteomics analyses are mostly based on published data^4^, available through ProteomeXchange at PXD004825, with the exception of those regarding larval samples described below.

#### Gel separation and protein digestion

Protein samples were loaded on a mini polyacrylamide gel and migrated about 2 cm. After Coomassie staining, regions of gel lanes containing visible bands were excised into 2–5 pieces, depending on the gel pattern. Gel pieces were digested with sequencing-grade trypsin (Promega, Switzerland) as previously described^43^. Extracted tryptic peptides were dried and resuspended in 0.1% formic acid, 2% (v/v) acetonitrile for mass spectrometry analyses.

#### Proteomic mass spectrometry analyses

Tryptic peptide mixtures were injected on a Dionex RSLC 3000 nanoHPLC system (Dionex, Sunnyvale, CA, USA) interfaced via a nanospray source to a high-resolution mass spectrometer based on Orbitrap technology (Thermo Fisher, Bremen, Germany): LTQ-Orbitrap XL or LTQ-Orbitrap Velos. Peptides were loaded onto a trapping microcolumn (Acclaim PepMap100 C18, 20 mm × 100 mm ID, 5 mm, Dionex) before separation on a C18 reversed-phase analytical nanocolumn at a flowrate of 0.3 ml/min.

The LTQ-Orbitrap Velos mass spectrometer was interfaced with a reversed-phase C18 Acclaim Pepmap nanocolumn (75 mm ID × 25 cm, 2.0 mm, 100 Å, Dionex) using a 65-min gradient from 5% to 72% acetonitrile in 0.1% formic acid for peptide separation (total time: 95 min). Full MS surveys were performed at a resolution of 30,000. In data-dependent acquisition controlled by Xcalibur software, the 15 most intense multiply charged precursor ions detected in the full MS survey scan were selected for HCD fragmentation (NCE = 40%) in the orbitrap at a resolution of 7,500, with an isolation window of 1.7 m/z, and then dynamically excluded from further selection for 25 s.

LTQ-Orbitrap XL instrument was interfaced with a reversed-phase C18 Nikkyo (75 mm ID × 15 cm, 3.0 mm, 120 Å, Nikkyo Technos, Tokyo, Japan) nanocolumn using a 90-min gradient from 4% to 76% acetonitrile in 0.1% formic acid for peptide separation (total time: 125 min). Full MS surveys were performed at a resolution of 60,000. In data-dependent acquisition controlled by Xcalibur software, the 10 most intense multiply charged precursor ions detected in the full MS survey scan were selected for CID fragmentation (NCE = 35%) in the LTQ linear trap with an isolation window of 4.0 m/z and then dynamically excluded from further selection for 60 s.

#### Proteomic data analysis

MS data were analyzed using Mascot 2.6 (RRID:SCR_014322, Matrix Science, London, UK) set up to search the UniProt (RRID:SCR_002380, www.uniprot.org) database restricted to *C*. *floridanus* (UniProt, December 2017 version: 14,802 sequences) and a custom database containing the re-annotated esterases (26 sequences). Trypsin (cleavage at K, R) was used as the enzyme definition, allowing two missed cleavages. Mascot was searched with a parent ion tolerance of 10 ppm and a fragment ion mass tolerance of 0.50 Da (LTQ-Orbitrap XL data) or 0.02 Da (LTQ-Orbitrap Velos data). The iodoacetamide derivative of cysteine was specified in Mascot as a fixed modification. N-terminal acetylation of protein, deamidation of asparagine and glutamine, and oxidation of methionine were specified as variable modifications.

Scaffold software (version 4.8, RRID:SCR_014345, Proteome Software Inc., Portland, OR) was used to validate MS/MS based peptide and protein identifications, and to perform dataset alignment. Peptide identifications were accepted if they could be established at greater than 90.0% probability as specified by the Peptide Prophet algorithm^44^ with Scaffold delta-mass correction. Protein identifications were accepted if they could be established at greater than 95.0% probability and contained at least two identified peptides. Protein probabilities were assigned by the Protein Prophet algorithm^45^. Proteins that contained similar peptides and could not be differentiated based on MS/MS analysis alone were grouped to satisfy the principles of parsimony and in most cases these proteins appear identical in annotations. Proteins sharing significant peptide evidence were grouped into clusters.

Quantitative spectral counting was performed using the normalized spectral abundance factor (NSAF), a measure that takes into account the number of spectra matched to each protein, the length of each protein and the total number of proteins identified in the experiment^46^. To compare relative abundance across samples with notably different protein abundance, we divided each protein’s NSAF value by the total sum of NSAF values present in the sample.

The additional larval proteomics data are available through ProteomeXchange at PXD009982.

### PCR-based annotation

Total RNA was extracted from four groups of four minor *C*. *floridanus* workers. Extraction was performed with RNeasy Plus micro kit and RNase-Free DNase Set (Qiagen) and reverse transcription with TAKARA PrimeScript™ RT Reagent Kit (Perfect Real Time). cDNA from four replicate samples were pooled and analyzed by PCR to determine whether Cflo.Est3 and Cflo.Est18 were part of the same gene. Sequences were edited and aligned (CLUSTALW) in MEGA v6.0. Primers, gel, and the resultant sequence of Cflo.Est18* can be found in Supplementary File 1.

### Gene annotation

The genomes and transcriptomes used in the phylogenetic analyses are listed in Supplementary File 1. Publicly available, curated carboxylesterase genes from these species were used as queries for re-annotating carboxylesterase genes using the tool genBlast^47,48^. In brief, genBlast aligns the protein query sequences to target sequences using BLAST, and stitches blast hits into complete gene structures while identifying splice junctions at exon/intron boundaries. To alias the resulting gene annotations to NCBI accession numbers, we used BLAST against the nonredundant (nr) database, followed by a synteny check. Based on these analyses, we propose new predicted gene-annotations (Supplemental File 1).

### Phylogeny reconstruction

Phylogenetic trees were built using RAxML version 8.1.15^49^, with the PROTCATLG model, and 100 bootstraps repeats (with the exception of Fig. 1, with 1000 repeats). Before running RAxML, predicted protein sequences of the annotated genes were aligned using the multiple sequence alignment program PRANK^50^. After using RogueNaRok (http://rnr.h-its.org/) to prune rogue taxa, the carboxylesterase genes of 39 species listed in Supplementary File 1 were used to build a preliminary phylogenetic tree (Supplementary Figure S3). The subtree containing the *A*. *mellifera* JHE and esterases in the *C*. *floridanus* trophallactic fluid (Fig. 2) were then separately aligned and their phylogeny reconstructed. In addition, to observe the trophallactic esterases in the context of phylogenetically related esterases whose functions are known, we built a tree of characterized JHEs, the re-annotated esterases of model organisms and six representative ants species (denoted ‘representative-tree’; Supplementary Fig. S4, included species are outlined in Supplementary File 2).

### Tests for positive selection

The ratio of non-synonymous to synonymous substitutions (dN/dS) was used to test for positive selection on genes belonging to the clade for which the *A*. *mellifera* JHE is basal  (the clade containing the *C*. *floridanus* trophallactic esterases) (Figure 2). Two species, *Nylanderia pubens* and *Myrmica rubra*, were removed due to poor assembly quality. Sequences that were redundant or shorter than 100 amino acids were filtered out before alignment. For analyses in Figs 2b and 3, sequences shorter than 400 amino acids were filtered out before alignment. Protein sequences were aligned using PRANK^50^, reverse-translated into codon alignment, and sites with alignment uncertainty were masked with a 0.5 score cutoff using GUIDANCE2 scores^51,52^. To test for positive selection, we used the modified branch-site test model A^53^, implemented in the PAML package, version 4.8a^34^. This is a likelihood ratio test (LRT) that compares a model allowing positive selection on one of the branches of the phylogeny to a model that allows no positive selection. LRT *p*-values for each branch were corrected into *q*-values to control the false discovery rate (FDR)^54^. Each branch with positive LRT at FDR <0.1 (Supplementary File 3) was labeled in red in Fig. 2b. Sites under positive selection were identified based on their posterior probability for dN/dS >1 in the branches that passed the LRT.

### Structure Analysis

Sequences were aligned to PDB 2FJ0, the *M*. *sexta* JHE^26^ using PROMALS3D REF. Chimera^55^ was used to visualize structures. The command swapaa was used to alter amino acids.

### Long-term development

To determine the effect of OTFP and exogenous JH on larval development, ants were taken from laboratory *C*. *floridanus* colonies to construct five replicates for each treatment condition. Approximately 90% of the ants were collected from inside the nest on the brood, while the remaining 10% were taken from outside the nest. Each colony explant had 20–30 workers (each treatment had the same number of replicates of any given colony) and was provided with five second- or third-instar larvae from their own colony of origin (staged larvae were equally distributed across replicates). Each explant was provided with water, 30% sugar water and maple-syrup-based ant diet. For each treatment both sugar water and food was supplemented with either solvent alone (ethanol), JH III, OTFP, or JH III and OTFP together (375 µM JH, 40 nM OTFP final concentration). Like JH, OTFP was diluted in ethanol (OTFP generously provide by Prof. Takahiro Shiotsuki, National Institute of Agrobiological Science). The low concentration of OTFP was chosen based on^27^ so as to inhibit only JHEs and not other enzymes. No insect-based food was provided. Food sources were refreshed twice per week. When JH and OTFP were administered in sugar water the solution was provided in a glass tube, and when on food, they were administered on a glass coverslip because both JH and OTFP are adherent to plastic.

Twice weekly before feeding, each explant was checked for the presence of pre-pupae, and developing larvae were counted and their length was measured using a micrometer in the reticle of a stereomicroscope. Upon pupation, or cocoon spinning, larvae/pupae were removed and placed in a clean humid chamber until metamorphosis. The head width of the pupae was measured using a micrometer in the reticle of a stereomicroscope 1–4 days after metamorphosis (head width is not stable within the first 24 h after removal of the larval sheath). Larval survival past metamorphosis was assessed at the same time of head-width measurement. Long-term development experiments were stopped when larvae had not changed in size for 1 week, which occurred after six weeks. Of larvae that did not undergo pupation during the experiment, ~85% were eaten by nursing workers.

### Sample sizes, data visualization and statistics

For long-term development experiments, the number of same-staged larvae per colony was the limiting factor for the number of replicates per experiment. Statistics were analyzed in R using built-in functions and ‘lmer’ and ‘glmer’ functions of the lme4 package, and mixed model p-values were calculated with the ‘lmerTest’ package. No data points were excluded as outliers and all replicates discussed are biological not technical replicates. Phylogenetic trees were visualized in FigTree 1.4.3. Molecular graphics and analyses were performed with the UCSF Chimera package^55^. Chimera is developed by the Resource for Biocomputing, Visualization, and Informatics at the University of California, San Francisco (supported by NIGMS P41-GM103311).

## Electronic supplementary material

Supplementary Information

Supplementary File 1

Supplementary File 2

Supplementary File 3

Supplementary File 4

Supplementary File 5

## Data Availability

All data generated or analyzed during this study are included in this manuscript, its supplementary files and through ProteomeXchange at PXD009982 and PXD004825.
