# Supplementary material for: Molecular evolution of juvenile hormone esterase-like proteins in a socially exchanged fluid
Source: Sci Rep. 2018 Dec 13;8:17830. doi: 10.1038/s41598-018-36048-1 (PMC6293014; doi:10.1038/s41598-018-36048-1)
Supplement: Supplementary file 1 — Supplementary Information [file 41598_2018_36048_MOESM1_ESM.pdf]

Supplementary Information

Molecular evolution of juvenile hormone esterase-like proteins in a socially exchanged fluid

Adria C. LeBoeuf*[1,2,3], Amir B. Cohanim[4], Céline Stoffel[3], Colin S. Brent[5], Patrice Waridel[6], Eyal Privman•[4], Laurent Keller•[3], Richard Benton•[2]

* Corresponding: adrialeboeuf@gmail.com
• These authors contributed equally

1. Department of Physics of Complex Systems, Weizmann Institute of Science, Rehovot, Israel
2. Center for Integrative Genomics, University of Lausanne, Lausanne, Switzerland
3. Department of Ecology and Evolution, University of Lausanne, Lausanne, Switzerland
4. Department of Evolutionary and Environmental Biology, Institute of Evolution, University of Haifa, Haifa, Israel
5. Arid Land Agricultural Research Center, USDA-ARS, Maricopa, United States
6. Protein Analysis Facility, University of Lausanne, Lausanne, Switzerland

This file contains:

Supplementary Table S1. Variable presence of JH in social insect trophallactic fluid.

Supplementary Table S2. Esterase abundance in different fluids and species.

Supplementary Figure S3. The phylogenetic tree used to define the clade containing the trophallactic esterases.

Supplementary Figure S4. Maximum likelihood gene tree of re-annotated esterases longer than 400 amino acids from 16 insect species.

Supplementary Table S5. Results of the branch-site test for the clade containing the trophallactic esterases.

Supplementary Figure S6. Branch numbers for trees in Figure 2.

Supplementary Figure S7. Branch length is correlated with protein abundance in trophallactic fluid.

Supplementary File Legends 1-5

Experiment 1

Species	JH III (pg/µl)	Colony
C. fellah	1815.44	C28
C. fellah	1356.92	C33
C. fellah	3621.84	C5
C. fellah	6787.81	C33
C. fellah	8142.26	C28
C. fellah	1624.9	C5
C. floridanus	19949.24	A13
C. floridanus	8712.29	A17
C. floridanus	9268.36	A23
C. floridanus	21779.98	A24
C. floridanus	5679.53	A26
C. floridanus	25299.61	A5
C. floridanus	6052.23	B52
C. floridanus	6888	A13
C. floridanus	1484.54	A17
C. floridanus	2895.08	A23
C. floridanus	4712.64	A24
C. floridanus	2981.26	A26
C. floridanus	12036.16	A5
C. floridanus	6131.24	B52

Experiment 2

Species	JH III (pg/µl)
A. mellifera	0.03
A. mellifera	0.01
A. mellifera	0.06
A. mellifera	0.03
S. invicta	0.58
S. invicta	0.23
S. invicta	0.1
S. invicta	0.06

Supplementary Table S1. Variable presence of JH in social insect trophallactic fluid. JH titers in trophallactic fluid pooled from groups of individuals from different colonies of *C. floridanus* (12-19 individuals, 1-12 µl), *C. fellah* (16-46 individuals, 10-12 µl), *S. invicta* (39-137 individuals, 1-9 µl) and *A. mellifera* (5-20 individuals, 11-35 µl). Each line corresponds to trophallactic fluid from only one colony but many individuals. Measurements of *Camponotus* trophallactic fluid were performed in a separate experiment from those of *S. invicta* and *A. mellifera* trophallactic fluid. The GC-MS peaks corresponding to JH in the *S. invicta* and *A. mellifera* experiment were not above the noise floor of the assay.

Name	Accession Number	*C. floridanus* Trophallactic fluid (n=15)	*C. fellah* Trophallactic fluid (n=6)	*C. floridanus* Hemolymph (n=6)	*C. floridanus* Larvae (n=2)
Cflo.Est6	E2ADW6_CAMFO	0%	0%	**< 0.1%**	**< 0.1%**
Cflo.Est8	E2ADW9_CAMFO	0%	0%	0%	**< 0.1%**
Cflo.Est9	E2ADW4_CAMFO	0%	0%	0%	0%
Cflo.Est10	E2ADW3_CAMFO	0%	0%	0%	0%
Cflo.Est11	E2ADW2_CAMFO	0%	0%	0%	**< 0.1%**
Cflo.Est12	E2A4N6_CAMFO	**0.1% ± 0.6%**	**0.2% ± 0.2%**	0%	0%
Cflo.Est13	E2A4N5_CAMFO	**0.6% ± 0.5%**	**0.8% ± 0.2%**	**0.12% ± 0.09%**	**< 0.1%**
Cflo.Est14	E2AI90_CAMFO	**2.0% ± 0.9%**	**0.6% ± 0.2%**	0%	0%
Cflo.Est15	E2AJL9_CAMFO	**0.5% ± 0.6%**	**1.0% ± 0.5%**	0%	0%
Cflo.Est16	E2AJL8_CAMFO	**13.0% ± 3.0%**	**0.4% ± 0.1%**	0%	**< 0.1%**
Cflo.Est17	E2AJL7_CAMFO	0%	**0.1% ± 0.2%**	0%	0%
Cflo.Est18*	E2ANU0_CAMFO + E2AJM0_CAMFO	**2.7% ± 1.2%**	**0.2% ± 0.1%**	0%	0%
Cflo.Est22	E2AM67_CAMFO	0%	0%	**0.34% ± 0.16%**	0%
Cflo.Est23	E2AM68_CAMFO	0%	0%	**0.76% ± 0.35%**	**< 0.1%**
Cflo.Est24	E2ADW8_CAMFO	0%	0%	**< 0.1%**	**< 0.1%**
Cflo.Est25	E2AG31_CAMFO	0%	0%	**< 0.1%**	**< 0.1%**
Cfel.Est1	comp44490_c0_seq1_2_217	0%	**0.8% ± 0.3%**	0%	0%
Cfel.Est2	comp45677_c0_seq1_3_767 (+2)	0%	**5.4% ± 1.5%**	0%	0%
Cflel.Est6	comp46324_c0_seq1_2_208	0%	**1.1% ± 0.3%**	0%	0%
Cfel.Est7	comp53526_c0_seq1_3_458	0%	**3.3% ± 0.4%**	0%	0%
Cfel.Est20	comp62625_c0_seq1_17_295	0%	**2.1% ± 1.2%**	0%	0%
Cfel.Est21	comp62625_c0_seq2_153_389	0%	**2.1% ± 1.3%**	0%	0%

Name	Accession Number	*A. mellifera* Trophallactic fluid (n=6)
–	A0A088A5D7_APIME / LOC552229	**2.1% ± 1.2%**

Name	Accession Number	*S. invicta* Trophallactic fluid (n=3)
Sinv.Est4	XP_011162808.1 / 751219447	**0.2% ± 0.2%**
Sinv.Est15	XP_011164602.1 / 751222706	**0.1% ± 0.1%**
Sinv.Est16	XP_011162491.1 / 751218861	**< 0.1%**
Sinv.Est25	XP_011169840.1 / 751232451	**0.1% ± 0.2%**
–	XP_011160598.1 / 751215387	**0.3% ± 0.5%**

Supplementary Table S2. Esterase abundance in different fluids and species. Percent of total NSAF corresponding to each protein. Aside from larval samples, all data are from[4]. Esterases found in *C. floridanus, C. fellah* and *S. invicta* trophallactic fluids are numbered for reference in this paper and accession numbers are given. Supplementary Figure S4 is a gene tree containing *C. floridanus, S. invicta* and other species' esterase sequences. The esterase observed in *A. mellifera* trophallactic fluid is not the functionally characterized *A. mellifera* JHE; rather this esterase is sufficiently distant that it was not included by genBlast. Because of the relatively poor quality of the *C. fellah* transcriptome, *C. fellah* trophallactic fluid proteomic data were also analyzed against the *C. floridanus* sequences. Many spectra were identified as matching the *C. floridanus* trophallactic esterases, indicating that the *C. fellah* transcriptome is missing many of these orthologs.

Supplementary Figure S3

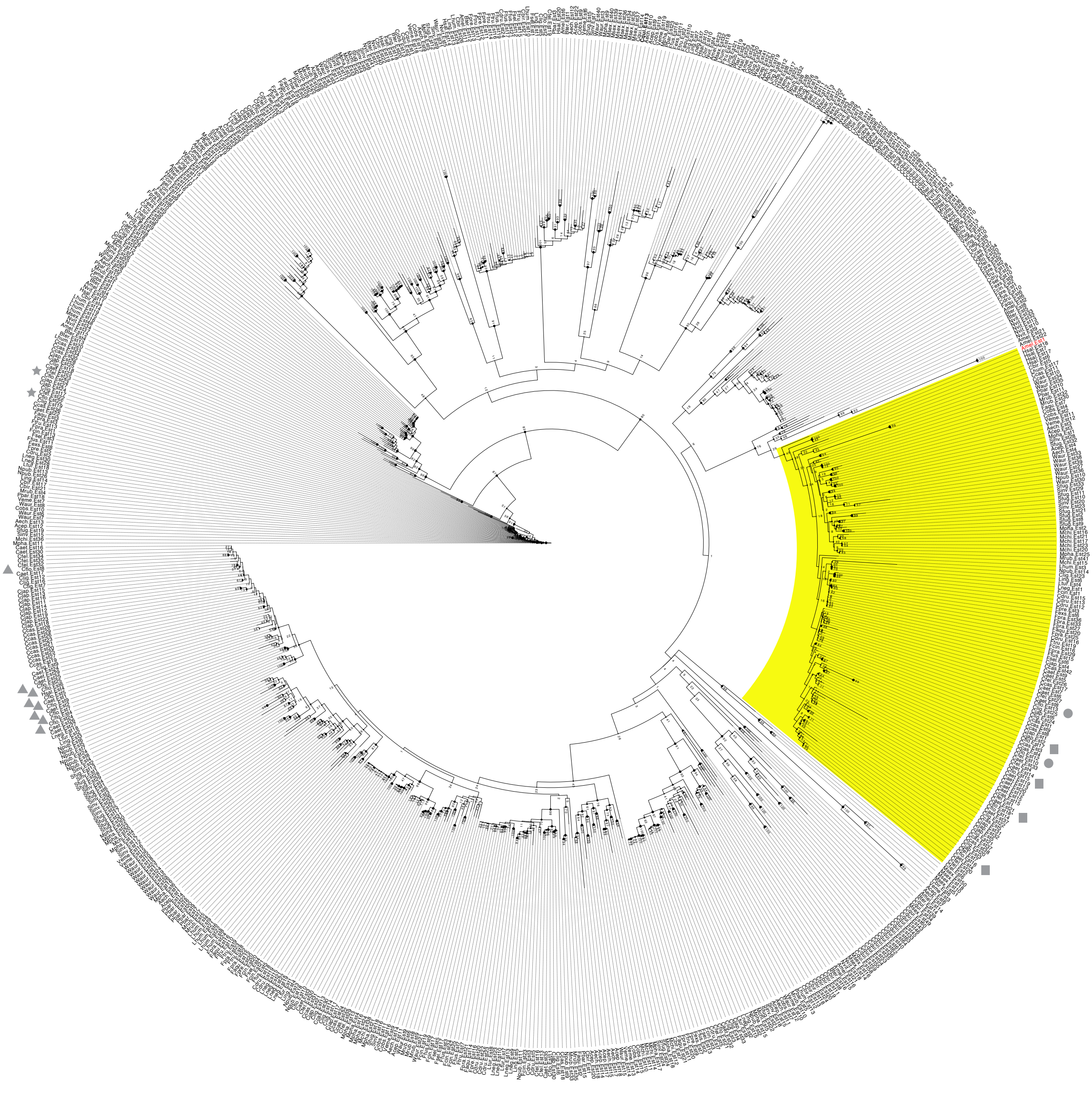

Supplementary Figure S3. The phylogenetic tree used to define the clade containing the trophallactic esterases. Sequences that were redundant or shorter than 100 amino acids were filtered out before alignment. The *A. mellifera* JHE, Amel.Est1, is indicated in red and the *C. floridanus* esterases in tandem arrays are indicated by symbols corresponding to their tandem arrays in Figure 1. The clade containing Amel.Est1 and the *C. floridanus* trophallactic esterases is highlighted in yellow. The relation between numbering shown throughout this paper and accession numbers can be found in Supplementary File 5.

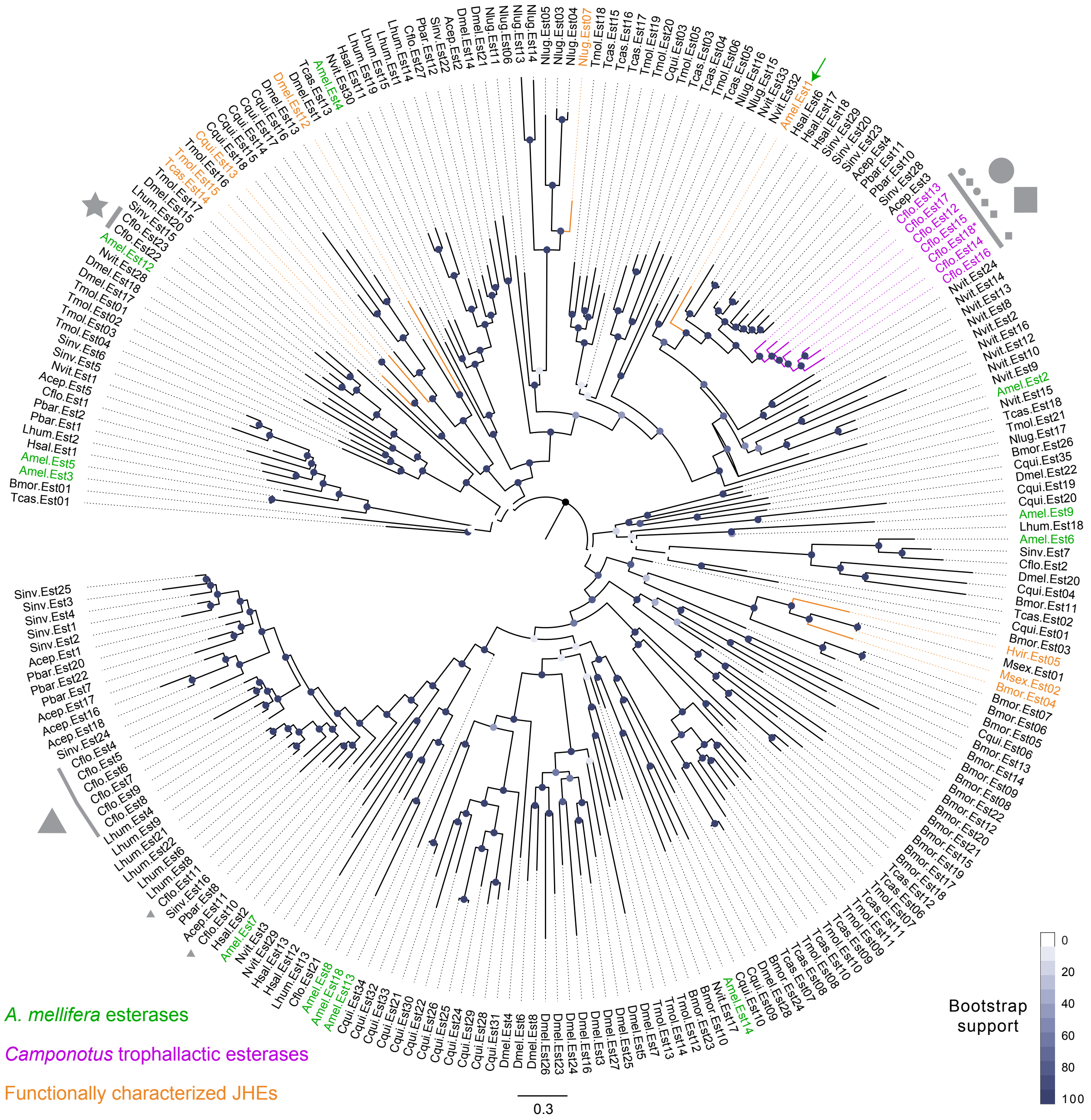

Supplementary Figure S4. Maximum likelihood gene tree of re-annotated esterases longer than 400 amino acids from 16 insect species. The tree is midpoint rooted. Functionally characterized JHEs are marked in orange. Purple indicates the *Camponotus* trophallactic esterases. *A. mellifera* esterases are indicated in green and with a green arrow for the *A. mellifera* JHE. Node color indicates bootstrap value from 0 to 100. Tandem arrays in *C. floridanus* are indicated by their symbols. Trophallactic esterases observed in *S. invicta* (Sinv.Est4, 15, 16 and 25) are found in the basal subclade containing the “triangle” *C. floridanus* esterases.

Selection analysis with sequences >400 aa

branch No.	W	p-value	q-value
**10**	999.00	1.3E-04	0.057
**105**	66.67	3.3E-05	0.057
**11**	78.36	7.2E-05	0.057
**14**	48.09	2.5E-03	0.057
**18**	10.53	2.5E-04	0.057
**2**	96.10	3.3E-05	0.057
**30**	61.38	2.2E-03	0.057
**5**	118.26	1.9E-02	0.098
**51**	207.69	1.6E-03	0.057
**52**	47.66	9.8E-04	0.057
**54**	95.27	3.3E-05	0.057
**55**	793.57	1.1E-04	0.057
**56**	134.31	3.3E-04	0.057
**57**	19.06	4.5E-04	0.057
**58**	5.57	1.0E-04	0.057
**62**	999.00	5.2E-03	0.061
**73**	998.99	2.9E-04	0.057
**96**	383.26	4.0E-04	0.057
**98**	4.84	2.0E-02	0.098
**99**	999.00	9.0E-03	0.069

Supplementary Table S5. Results of the branch-site test for the clade containing the trophallactic esterases. The displayed branches are inferred to be under positive selection based on the LRT of the branch-site test with FDR adjusted p-value (q-value) < 0.1. Results are shown for analysis for >400 amino acids sequences.

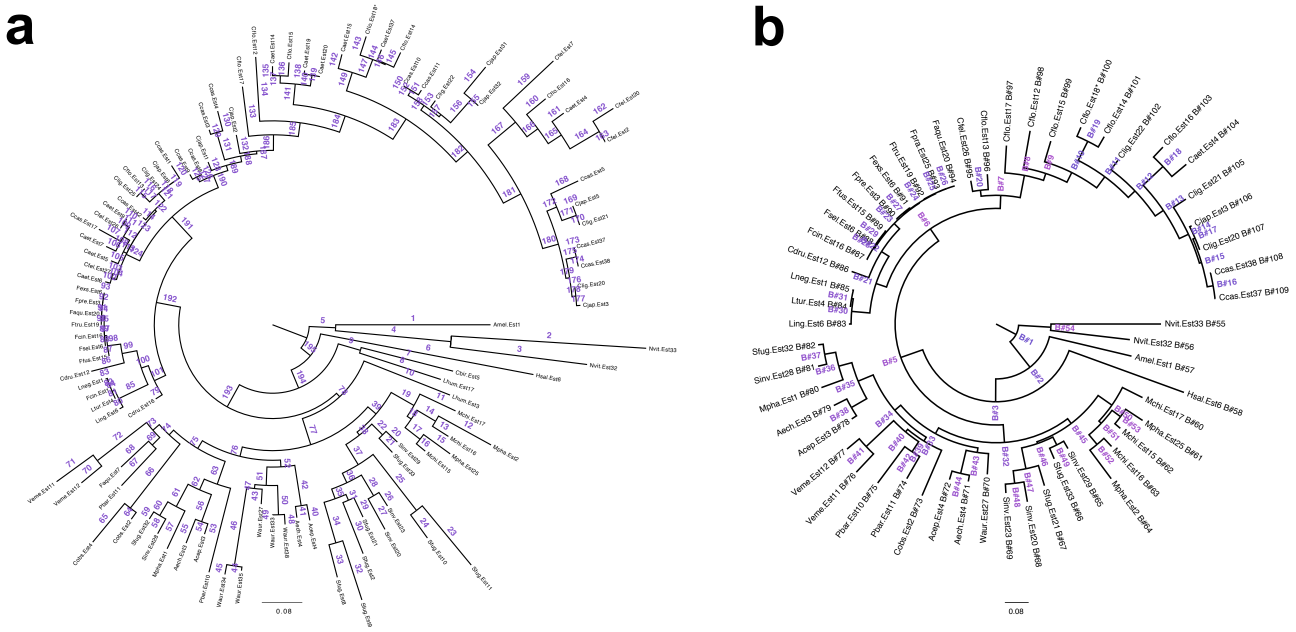

Supplementary Figure S6. Branch numbers for trees in Figure 2. (a) Clade containing 101 esterases from 31 species of ants, *A. mellifera* and *N. vitripennis* (sequences >100 amino acids); (b) Clade containing 54 esterases from 29 species of ants, *A. mellifera* and *N. vitripennis* (sequences >400 amino acids). Branch numbers are indicated in purple, and branch significance values for (b) can be found in Supplementary Table S5.

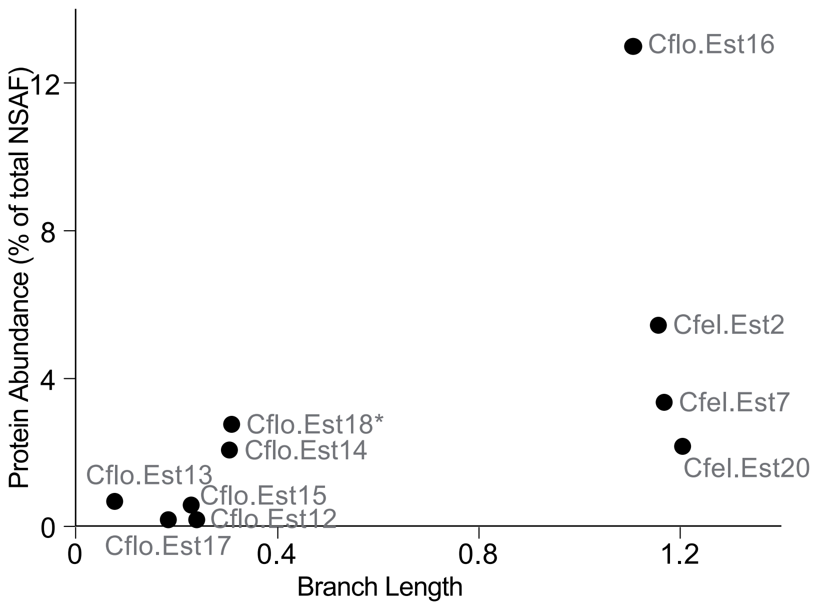

Supplementary Figure S7. Branch length is correlated with protein abundance in trophallactic fluid. Cumulative lengths for branches after branch #191 in Supplementary Figure S6a for each of the esterases measured in trophallactic fluid and the protein abundance for each. Spearman's rank correlation $p < 0.0005$, $\rho=0.85$.

Supplementary File Legends

Supplementary File 1. Zip-file of all esterase annotations, and the RT-PCR based determination that E2ANU0 and E2AJM0 (Cflo.Est3 and Cflo.Est18) are the same gene.

Supplementary File 2. Species used to build the three phylogenetic trees in Supplementary Figure S3, Supplementary Figure S4, and Figure 2. Species present in Supplementary Figure S3 but not Figure 2 were excluded due to suboptimal assembly quality.

Supplementary File 3. Positively selected amino acid changes in the abundant trophallactic esterases. Amino acid changes are listed for each of the positively selected branches. Amongst these, those that were significant according to the Bayes Empirical Bayes (BEB) analysis are indicated with an asterisk. The second sheet shows an alignment of the sequences in Figure 2b. The third sheet displays the amino acid changes shown in Figure 3.

Supplementary File 4. Source data for Figure 4.

Supplementary File 5. The relation between numbering shown throughout this paper (Gspe.EstX) and available accession numbers. Where no accession number is listed, the sequence can be found in our annotations (Supplementary File 1).
