# Supplementary material for: Molecular evolution of juvenile hormone esterase-like proteins in a socially exchanged fluid
Source: Sci Rep. 2018 Dec 13;8:17830. doi: 10.1038/s41598-018-36048-1 (PMC6293014; doi:10.1038/s41598-018-36048-1)
Supplement: Supplementary file 2 — Supplementary File 1 [file 41598_2018_36048_MOESM2_ESM.zip › our-gene-annotations/SI Assembling CfloEst18-3.pdf]

E2ANU0 and E2AJM0 (Cflo.Est3 and Cflo.Est18) are pieces of the same transcript lying on the ends of two different contigs. Primers were designed to amplify the intervening sequence between these two fragments upon RT-PCR of whole-body cDNA merged from four separate extractions. The positive control of a region of Cflo.Est16 (E2AJL8) is also shown.

Primers	Sequences
**NU0JM0-R**	GTGAAAGGAGTGATAAGATTGTAAAAG
**3031-F**	GAGGGTGCCGAAGATTGCTT
**3033-F**	GCTACGAAATTCGGTTCTCCG
**1473-R**	CAACAACTGGTCCGAAAGGAG

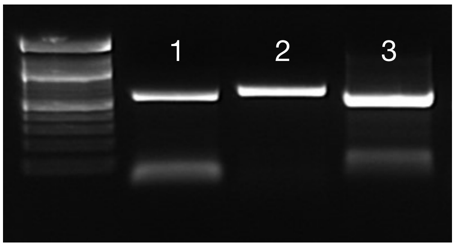

Primer Pair	Target	
**1**	Cflo.Est3 / Cflo.Est18	3031-F/NU0JM0-R
**2**	Cflo.Est3 / Cflo.Est18	3033-F/NU0JM0-R
**3**	Cflo.Est16	3031-F/1473-R

Intervening sequence for Cflo.Est3 / Cflo.Est18 aka E2ANU0 / E2AJM0 from Primer Pair 2 (3033-F/NU0JM0-R):
TGCATTCAATATGATCAATTTGCTTCCGATTCTGCCGATAAAGTCGAGGGTGCCGAAGATTGCTTGTATTTA
AATATTTATGTACCAGTACGAAACAAGACGGAAAACAAGACATCTATGCCAGTGTTGTTTTGGATCCACGG
TGGCGCTTTTCAATATGGTAGCGGTATGATTTATGGAGCCACATATTTGATGGATAGCGACGTCATACTCGT
CACATTTAACTATCGATTGGGACCGATGGGTTTTCTTAGCACGGAAGATGAGGTAGTTCCTGGCAACATG
GGTCTAAAAGACCAAAATATGGCACTTCGCTGGGTATTTCAgAATATCGAGTCGTTCGGTGGCGATCCAAA
CGGAATAACTTTATTTGGTCAGAGTGCTGGTAGTGCAAGTGTGCATTATCACTACTTATCGCCAATGAGCG
CAGGACTCTTTCGAGGAGGAATATCGTACAGCGGAACAGCGTTCGATTGTTGGGCGCAAACTGAGAATT
CTTTAGAGAAAACTAAAAAACTGAGCGCTTTAATGGGATGTCCTACAACTAATTCTAGAGACATGATAGATT
GCTTGAGACATCGACCGGCTCGAGATATCGTACAATGCCAATATTCTACAAACGAATTTATGTATTT

Full sequence for Cflo.Est18*:
GCCAATTCGCAATGATTTCTGCAAAGGATAGCACGTTACTGAATACCATGTTCATAAGACTTCTGATCGTT
CTGCTTTGTTTAAACTTAACCATACTAGCAAATTCAGAGGAGATTGCACCAAAAGTTAAAACTCTCTCAGG
AGCACTAAAAGGCTATTATAAAATATCACAGTATGGCAGAAAATACGAAGCATACGAAGGGATTCCTTATGC
GTTACCACCTATTGGAGAATTAAGATTCAAGCCTCCTCGACCAATAACGCCATGGATCAGCGAACTATCGG
CTACGAAATTCGGTTCTCCGTGCATTCAATATGATCAATTTGCTTCCGATTCTGCCGATAAAGTCGAGGGT
GCCGAAGATTGCTTGTATTTAAATATTTATGTACCAGTACGAAACAAGACGGAAAACAAGACATCTATGCCA
GTGTTGTTTTGGATCCACGGTGGCGCTTTTCAATATGGTAGCGGTATGATTTATGGAGCCACATATTTGAT
GGATAGCGACGTCATACTCGTCACATTTAACTATCGATTGGGACCGATGGGTTTTCTTAGCACGGAAGAT
GAGGTAGTTCCTGGCAACATGGGTCTAAAAGACCAAAATATGGCACTTCGCTGGGTATTTCAGAATATCG
AGTCGTTCGGTGGCGATCCAAACGGAATAACTTTATTTGGTCAGAGTGCTGGTAGTGCAAGTGTGCATTA
TCACTACTTATCGCCAATGAGCGCAGGACTCTTTCGAGGAGGAATATCGTACAGCGGAACAGCGTTCGAT
TGTTGGGCGCAAACTGAGAATTCTTTAGAGAAAACTAAAAAACTGAGCGCTTTAATGGGATGTCCTACAA
CTAATTCTAGAGACATGATAGATTGCTTGAGACATCGACCGGCTCGAGATATCGTACAATGCCAATATTCTA
CAAACGAATTTATGTATTTCTTTTACAATCTTATCACTCCTTTCACACCAGTAGTTGAAAAAGGCAGTGATA
CACCTTTCATCGATAAGACACCAGTTGAAATCGTGAATAACGGTGACGTACAAGATTTACCTTGGGTTACA
AGTGTAGTGAGTCAAGACGGCCTTTATCCCGTAGCTGAATTCATCGCTGACAATGAAACTCTGAAACAATT
AAACAACAACTGGGATCATCTTGCTCCGCGTTGCCTAGACTTTTATGACACCATCCCAAAAGAGAAATAC
GTCGAAATTTCTCACATTATCAAGAAGCATTATTTCGGTACGAAACCAATAGACCAGACAACTACAAGCCA
ACTGGTACAATTGGCGAGTGACCGCTTCTTCTTTGTTGACAGTAAAAAGGCTGCGCTAATGCAAGCCAAA
GTAAATAAGAATCCAGTCTGGTATTCTTATTTTTCGTACAGAGGAAATCAAAGTTTAAGTGAATATTATAGCG
GTACAACTAATAATTACGGTGTCTGCCATGGCGATGATATAACATATATATTAGATACTCCTTGGGTGGATCC
AACAACAACGCAACGAGATCGCGATATGCAGAAACTCTTAATCGACTTTTGTGTATCTTTTGCTACAAATG
GAATTGCAAAAGTGTATGGTGCAAATTGGTCGCAATTAAATCCTTCAGAGAAGGAATTTCATTATTTGCATA
TTGCAAGTCCTACTGAGATTAACACGGGTAGCAATGCTGATTTCGGGGATGAAACATTCTGGAATTCACTT
AAGTTTAACGAGAATGTGTTGACCACGTAAAGAAATTGAGAAGAAGTGCTTTGAATGATTCTACGAGGAAA
TTCGACTCTTTCATCTATATTTACGCAACTAATTTTT
